# Regulating advance decision-making: potential and challenges for Malaysia

**DOI:** 10.1007/s41649-019-00078-2

**Published:** 2019-03-22

**Authors:** Hui Yun Chan

**Affiliations:** The Law School, Huddersfield Business School, University of Huddersfield, Huddersfield, UK

**Keywords:** Advance directives, Patient care, Autonomy, Malaysia, Decision-making

## Abstract

The right to refuse treatment is generally accepted in the legal and bioethics discourses; however, the use of advance directives remains contentious. Some jurisdictions have introduced statutory frameworks to govern the creation and implementation of advance directives, underpinned primarily by the recognition of respect for personal autonomy. Although there are no legislation and judicial decisions on advance decision-making in Malaysia, the considered view is that healthcare practitioners perceived its utility in managing patient care. This paper examines the potential and challenges of applying a statutory framework in Malaysia, drawing from relevant regulatory examples. It argues for greater public awareness within the healthcare discourse and knowledge dissemination regarding the availability, usage and clinical guidance on advance decision-making. The main conclusion drawn from this exploratory analysis is that further understanding of and education about advance decision-making within the population and healthcare practitioners should precede the implementation of a statutory regime in Malaysia.

## Introduction

Advance directives (ADs) provide the opportunity for people to express their treatment preferences which will become effective when the person is unable to refuse consent at a future time. ADs refusing treatment is generally controversial, although the right to contemporaneous treatment refusal is commonly accepted, as long as the patient is informed and has the requisite mental capacity to refuse any proposed treatment.^1^ The use of ADs as a means of governing end-of-life care is not new, but introducing the topic in the context of a multicultural population raises both ethical and legal challenges. The lack of clarity regarding the availability, legal status and clinical guidance of ADs generates uncertainty in clinical practice. This uncertainty prompted some jurisdictions to legislate AD laws. Some common law examples from Singapore, Australia and England reveal an approach that favours regulation in governing the use of advance decision-making. Should Malaysia adopt a similar legislative option? It is on this basis that this article examines examples from relevant statutory models and explores their potential and challenges in Malaysia. Such a consideration is premised upon the view that it is timely to consider the subject matter with the growing concerns raised by healthcare practitioners (Gendeh et al. 2016). While this paper is focused on the Malaysian context, the analysis has broader implications to other developing Asia and Southeast Asia jurisdictions that are considering proposals towards implementing AD laws.

Section A has provided an overview of the issues that will be dealt with in the paper. Section B outlines the legal and ethical challenges with making ADs, drawing from the English and Singapore examples where statutory frameworks govern their use. Some interim observations are proposed and contrasted with the Malaysian setting. Section C delves into the potential and challenges of developing an AD law in Malaysia. It highlights the extent of AD awareness within the population and influences of faith and familial decision-making models on AD practices, with reference to Malaysia’s cultural diversity. Section D concludes with the proposal to advance an understanding of ADs through promotional efforts in order to increase awareness of ADs as a way forward.

## Regulating advance decision-making

### Overview

Malaysia inherited its legal system from England and common law cases are highly persuasive. In the rich area of medical negligence, principles derived from the common law are highly persuasive and adapted to suit domestic situations.^2^ The social demography is depicted by an ethnicity of Malay, Chinese and Indian as the predominant ethnic groups, while also encompassing native populations in the eastern part of the country (Department of Statistics Malaysia 2016). While there is no law in Malaysia on the topic, it is postulated that in the absence of a specific legal framework on ADs, the common law right to refuse treatment applies (MMA 2017). The Malaysian Medical Association (MMA) equally affirmed a patient’s right to refuse treatment, when the patient is adequately informed (MMA 2017). This connotes that as long as the person is mentally competent when the refusal is expressed, with full understanding of the nature and consequences of the refusal, the refusal will be respected.

The Malaysian Medical Council (MMC) guideline on consent for treatment is in line with common law principles; that a mentally competent person can refuse treatment even if the reasons for such refusal are deemed to be unwise, unknown or without any basis (MMC 2017). Additionally, the MMC recommended that a patient’s refusal be recorded in writing, signed and dated by the patient. While ADs for treatment refusal are accepted, ADs requesting for termination of life are prohibited, similar to many jurisdictions. Paragraph 18 of the MMC guideline specifically addressed the application of ADs in clinical practice, particularly advising doctors not to act contrary to clearly written ADs. However, the guideline recommended doctors to consider the validity and applicability of the ADs where such ADs exist. Where doubts exist as to the validity of the ADs, treatment can be given in the event of an emergency. The MMC advised doctors to confer with the patient’s family members or to seek the assistance and guidance of fellow doctors where doubts about the scope and applicability of the AD arise (MMC 2017). This recommendation sheds light on how doctors may navigate treatment decisions in the light of doubtful ADs.

### Statutory regulation as an option to govern the use of ADs: legal and ethical challenges

An option that is often canvassed to regulate the use of ADs is through the statutory regime, exemplified by England and Wales, Australia and Singapore. The statutory argument for regulating the use of ADs normally appeals to patients’ rights to consent or refuse medical treatment underpinned by the popular notion of autonomy. A statutory framework arguably provides clarity, in terms of determining what constitutes a valid AD, while recognising the importance of patient autonomy in expressing their treatment preferences. The latter is especially helpful in jurisdictions or societies where there exists an awareness of the concept of patient rights. Regulation also serves as an impetus for compliance, for example in the case of clinicians who would be more aware of the importance of taking into account patient wishes in ADs once the law attaches legal significance to ADs. Conversely, it may mean that doctors may not necessarily comply with ADs in the absence of legislation for ADs (Tiano 2012).

Pursuant to this approach, specific and formal requirements are set out in the statute; addressing a range of conditions where ADs are accepted as valid. The regulatory framework may provide for the use of specific forms as part of the formality for an AD to be considered as valid, in addition to the requirements of mental capacity, voluntariness and understanding in creating the AD. Therefore, while an AD can be made verbally or in writing under the common law, the statutes may require that an AD be written, or to record verbal ADs into written format. Questions about subsequent changes are also addressed in the statutory regime, such as changes in the personal circumstances, medical advancements affecting treatment decisions and change of minds following the making of the AD.

As an illustration, the English Mental Capacity Act (MCA) 2005 prescribes specific requirements for ADs in Sections 24 to 26 that have to be complied with in order to be valid and binding, for example only an adult of 18 years and who possesses the mental capacity to decide can make ADs refusing treatment at a future time (MCA 2005 s24(1)). Additionally, the MCA specifies that an AD does not apply where the treatment is not the one specified in the AD, or if there are reasonable grounds to believe that circumstances exist which the person failed to anticipate at the time of making the AD and which would have affected his/her decision had he/she anticipated them (MCA 2005 s25(4)). In the case of life-sustaining treatment, the AD applies if the patient has verified that it applies at all cost and written, signed and witnessed (MCA 2005 s25). If those formal requirements are not met, then the AD does not bind the doctors.

While regulation provides clarity in procedures and implementation purposes, there are several limitations with the statutory approach (Orentlicher 1994; Willmott 2007; Castillo et al. 2011; Cartwright 2013). Primarily, statutes may unintentionally undermine common law ADs which do not comply with the formal requirements prescribed in the law. For example, where the law requires an AD to be written, signed and witnessed or in a specified form, failure to adhere to these requirements means that the AD would not be binding on healthcare professionals (Meisel et al. 2000; MCA 2005; AMDA 1996). The MCA example clearly illustrates that there are pre-requisites for ADs refusing life-sustaining treatment which affect their implementation. These requirements in turn necessitate knowledge and information regarding how to create legally compliant ADs. Additionally, further restrictions on the scope and language of the ADs have modified the way they are made under the common law. It can be reasonably anticipated that the general population may be unaware of these requirements, and without legal training or knowledge, they may not realise that the ADs would not be accepted due to technical inadequacies.

Some statutory framework, such as that introduced in Singapore requires ADs to be registered in order to be valid and binding. The ADs envisioned under the Singapore law only applies in terminal illness, with certification from doctors under limited circumstances. The strict application and narrow scope is a slight departure from conventional ADs, which are primarily used to refuse unwanted medical treatment. This approach could be attributed to its doctor-centred approach and strong familial decision-making culture which give rise to watertight provisions (Foo et al. 2013; Singapore Select Committee 1996). Since the introduction of the Singapore Advance Medical Directive Act (AMDA) in 1996, advance care planning has yet to become commonplace (Ng 2009; Menon 2012). Existing practices of doctor-family collusion in healthcare decision-making and that it is a criminal offence if a healthcare practitioner were to ask patients if they have ADs are among the factors that shape its development (Menon 2012).

Its usage is further compounded by the recognition that autonomy, in principle, has failed to flourish in a family-centric society (Radha Krishna et al. 2015). Although Singapore doctors are generally supportive of the AD concept, a study revealed that they were not unanimous about legislating ADs, preferring doctors to decide for patients (Tee et al. 1997). A low level of awareness regarding ADs, advance care planning and cultural taboo concerning end-of-life conversations similarly contributed to the difficulties in implementing patient preferences (Ng et al. 2013). In this regard, the principle of autonomy which underpins the exercise of ADs presents further challenge for implementation. While the law adopts the prevalent interpretation of autonomy in decision-making, this notion appears to be incompatible with its established socio-cultural practices. In this sense, the challenge faced by Singapore is similar to Malaysia and it is essential to re-evaluate the idea of introducing a legal framework underpinned by ethical concepts that are unsuitable to a particular social demography. Assuming that a similar legal framework as MCA or AMDA is introduced in Malaysia, it would be unsurprising that the Malaysian population would face comparable challenges as Singapore in accessing and successfully implementing ADs.

The ethical issues associated with ADs continue to be challenging and as illustrated above, the challenges are more likely to be amplified in multicultural populations with a plurality of values, beliefs and medical decision-making models (Giger et al. 2006; Kuczewski 1996; Payne et al. 2005; Sass et al. 1996; Winzelberg et al. 2005; Johnstone and Kanitsaki 2009). Autonomy features primarily in a medical law framework and is understood as respecting a person’s right to his/her own views and actions, including taking steps to encourage the person to act autonomously (Beauchamp and Childress 2013). This conception of autonomy may be incompatible with the model of healthcare decision-making in the Malaysian context.

Several important considerations thus arise in applying the AD concept in multicultural Malaysia. First, the recognition of patient autonomy, as understood in contemporary bioethics discourse, is still at a nascent stage in Malaysia. The majority of the population are more inclined towards collective decision-making within family circles. Secondly, healthcare practitioners are generally highly regarded by society as the decision-making authority concerning patient care and treatment (Qidwai 2005; Barilan and Weintraub 2001). Such deference to medical opinions is amplified by an embedded socio-cultural norm premised on strong familial-oriented values. Thirdly, the healthcare system in a developing country such as Malaysia is focused on primary healthcare services in curing the population, saving lives and reducing mortality from the burdens of diseases, which differs from the preventative medicine approach in more matured jurisdictions. Consequently, it can be inferred that the notion of creating and formalising ADs as a means of expressing treatment preferences may be perceived as healthcare privileges rather than as essential components of good patient care. It is perhaps unsurprising that the lack of information and awareness about ADs may have contributed to an inadequately prepared healthcare system that incorporates the provision of ADs as part of its healthcare delivery.

The legal challenges presented by ADs signify that statutory regulation and the ways in which it transforms how ADs are made have unintentionally impeded rather than supported their use. There remains a gap between an actual understanding of the AD-making process by the population and healthcare practitioners and their implementation according to the law. Questions such as what the restrictions or limitations of their use are or the extent to which healthcare practitioners know about the implementation of ADs in clinical settings remain open. The English example revealed that 10 years post implementation of the MCA, there was a low uptake of ADs and the law’s influence on healthcare decision-making remained subtle (Alghrani et al. 2016). Although there is an inclination to follow the developments in England and Wales due to inherited common law traditions, it would be prudent to examine their applications in the local context prior to introducing a similar legislative model. The ethical challenges meanwhile encompass an appreciation and practice of autonomy as understood within the local context. While there exists pockets of differences between diverse segments of the population (owing to varying economic incomes and educational levels), practicing autonomy through ADs remains challenging in reality due to the reasons outlined above. The following section considers the appropriateness developing an AD law for Malaysia, considering the various challenges and potential.

## Developing an AD law for Malaysia?

### Challenges and potential

Should Malaysia develop a statutory framework to govern the use of ADs in Malaysia? Although there are no legislation and judicial decisions on ADs in Malaysia, the considered view is that healthcare practitioners perceived their utility in managing patient care (Lai et al. 2016; Gendeh et al. 2016; Naidu 2017). Additionally, a recent questionnaire has been developed to assess the awareness and attitude of older people in Malaysia towards advance care planning, and to ascertain the difference of perceptions, if any, of various ethnic groups towards advance care planning (Lai et al. 2016). The authors postulated that this study would assist with the decision whether to legislate AD laws in Malaysia. (Lai et al. 2016).

It is anticipated that where such a wide-scale study is piloted in Malaysia, it would offer the opportunity to determine the practical and clinical concerns regarding ADs at the societal level. This would provide the chance to establish the awareness and receptiveness of the population towards making ADs. For example, an exploratory qualitative study within a geriatric unit in a Malaysian hospital on ADs and the concept of advance care planning found that the lack of knowledge about ADs did not discourage the elderly study participants from being receptive towards ADs (Htut et al. 2007). It is illuminating that while they recognised the importance of advance planning for medical treatment, formalising written ADs is considered as inessential, principally driven by their respective religious beliefs that one’s future is best left to fate or God. The investigators of the study recommended that doctors initiate end-of-life care conversations with the patient and their families to promote awareness of ADs among the population. A finding published 10 years later in a different study conducted on nursing home residents revealed that although about 70% of the elderly population surveyed were receptive to the AD concept, the prevalence rate is low (Koh et al. 2017). The authors suggested that religious beliefs and preferring the opinions of families and healthcare providers over their own influenced their decisions to have ADs (Koh et al. 2017). The authors came to a similar conclusion as Htut et al. (2007) that raising public awareness through primary care services and campaigns would encourage more people across the population to be aware of ADs (Koh et al. 2017). These studies, albeit limited, appear to highlight religious and cultural ideas as influential factors underlying their decisions to make ADs and the significance of creating AD awareness in the population.

Recent reports echoed the importance of advance care planning and the need for AD laws as a means of affirming and respecting a person’s treatment wishes (Jahn Kassim and Alias 2015, 2017; Oh 2018). The MMA President emphasised the need for guidance in supporting doctors to manage patient expectations and navigate family conflict in treatment decisions, citing a lack of supportive environment within clinical practice for patients to express their treatment preferences (Naidu 2017). While the Malaysian Code of Medical Ethics points towards the need to consider ADs and family wishes of dying patients, the low level of AD awareness and the lack of practices where doctors enquire about ADs makes it difficult for such compliance (Naidu 2017).

Tan Kiak Min’s (2018) recent article explored the option of relying on ethical codes and guidelines to guide advance decision-making in the absence of a specific AD law in Malaysia. Tan identified the problematic ways in which these ethical guidance and medical guidelines pose to their implementation, particularly the difficulty in determining the validity of ADs under the MMC guideline on consent for treatment of patients and where only written ADs would be considered. Tan appears to support the introduction of AD laws as a way to enforce the guidelines to ensure doctors’ compliance with the law. As highlighted in Section B I above, the MMC guideline advised that medical practitioners should refrain from providing treatment or performing any procedure where there is an unequivocal written directive by the patient that such treatment or procedure is not to be provided in the applicable circumstances. An immediate challenge presents itself: the current lack of understanding of ADs means that patients with valid written ADs, if any, remains a rarity. Further, the recommendation that doctors consult family members or colleagues where doubts exist about the AD means that ADs are often questioned and there exists an uncertainty where family members are unable to find such ADs or uphold the wishes of the now incapacitated patient. Thus, while legislation is an option, it would likely become valuable where there is greater awareness among the public and healthcare practitioners about ADs and a system in place to ensure its effective implementation.

While Tan Kiak Min (2018) rightly argued for coordinated efforts in building a practical AD framework for Malaysia, it is unclear how such a framework would look like. Nationwide consultations on ADs provide the opportunity to assess the preparedness of the legal, societal and healthcare systems in providing an efficient environment with appropriate resources to introduce and implement ADs. The legislature could then take steps towards consolidating and clarifying various existing code of ethics and guidelines and communicate these information through healthcare services. As gestured above, adherence to formalities under AD laws presumably require people who wished to make ADs to be aware of such legal requirements, as non-compliance undermines their application. This would necessitate an active approach on people to seek information on making ADs as well as a concerted effort by health or public authorities in promoting, raising awareness and disseminating information on ADs. As we have seen from the English example above, there are particular challenges with complying with a valid AD refusing life-sustaining treatment. While the English population may generally be more familiar with the concept of ADs compared to Malaysia, it does not necessarily follow that they are aware of the legal complexities in creating valid ADs. Additionally, the variation in interpreting and applying the ethical principle of autonomy in the two jurisdictions makes it unclear if introducing AD laws would make a difference in ensuring that wishes are respected.

Multiculturalism and religious beliefs are often cited as distinctive challenges in introducing and implementing end-of-life care planning in jurisdictions with diverse ethnicities (Johnstone and Kanitsaki 2009; Zager and Yancy 2011; Gendeh et al. 2016; Menon et al. 2018). Globalisation and migration have changed the population landscape, resulting in more diverse populations in predominantly Western countries, for example Australia and New Zealand. This entails a tailored approach to developing an AD framework that would function well within various segments of the population. New Zealand illustrates an inclusive approach in developing guidance for healthcare practitioners working with multicultural and multi-faith Asian patients and their families (Lim et al. 2012). Doctors are advised to consider the different interpretations of autonomy, non-maleficence and beneficence when discussing ADs with patients, and encouraging deeper discussions to discover patient values and facilitate their understanding of resuscitation orders through effective use of interpreters to aid with understanding English materials on ADs (Lim et al. 2012). Multiculturalism does not automatically imply detriments to introducing ADs; rather, it signifies that a modified approach is needed to accommodate patient needs for a successful implementation of AD discussions. This approach would very well apply to healthcare practitioners and their patients in Malaysia who come from diverse backgrounds professing various faiths.

The challenges of introducing AD laws in Malaysia ranged from knowledge deficiency among the population generally to an absence of consistent effort in advocating its use as part of healthcare delivery and existing models of familial healthcare decision-making. Nevertheless, there is potential in generating awareness through health advocacy as exemplified by the limited studies undertaken in the local context. The lessons drawn from England and Singapore offered insights into the effects of implementing statutory ADs, and demonstrated likely scenarios if AD laws were introduced in Malaysia now. The following section examines the option of non-statutory form and the ways in growing greater awareness among the population and healthcare practitioners.

### Maintaining the status quo and promoting ADs

The question whether to introduce legislation to govern the use of ADs or to leave it unregulated has been considered by the Law Reform Commission (LRC) of Hong Kong in 2004. The LRC, following an extensive consultation through its sub-committee, recommended that the concept of ADs should be promoted through non-legislative means, preferring to leave the option open until more people become aware of it (LRC 2004, 2006). This view is shared by the Hong Kong Food and Health Bureau through its Consultation Paper on the Introduction of the Concept of Advance Directives in Hong Kong in 2009.

This hesitation stems from the understanding that ADs remain a relatively new concept (Chung et al. 2017) and the LRC recommended the launching of public awareness activities to promote the concept of ADs, channelled via the Department of Health and District Offices. Other promotion efforts include garnering the support from the local medical council, medical association, the law society, hospital authorities, clinics, religious and communities groups in disseminating information on ADs. Meanwhile, people wishing to use ADs are encouraged to utilise the model form proposed by the LRC.

The recommendations of the LRC report have generated responses among some healthcare practitioners in Hong Kong (Tse 2010; Hui 2010). Some viewed AD laws as helpful in clarifying its legal status, similar to the English position, while retaining the flexibility as to its execution (Tse 2010). Clinical guidelines would help in the interim to address the implementation of ADs in clinical practice. Echoing similar views, Hui (2010) challenged the reasoning for not introducing AD laws in Hong Kong, arguing that the lack of awareness and the taboo of discussing ADs would similarly extend to discussions on advance care planning. Instead, Hui (2010) perceived the lack of preparedness of and engagements among healthcare practitioners in issues of death and dying in the societies have led to the current state. Hui (2010) further argued that time constraints further complicate the problem, as discussing ADs requires time.

It is clear that socio-cultural consideration where talking about ADs and death is taboo, combined with a preference for familial decision-making and a general lack of awareness regarding ADs and future care planning concepts are the key challenges in Hong Kong. Both considerations are similar to Malaysia; thus, the argument that it is premature to introduce AD legislation in Hong Kong would similarly hold true in Malaysia. While the Malaysian population is ethnically more diverse compared to Hong Kong, the various ethnic groups view family support as central in healthcare decision-making (Con 2007; Khanth 2006; Ng 2009). A point of difference however lies in the progress in promoting ADs to the public in increasing their awareness of ADs. It is unclear from the literature thus far that similar advocacy activities to raise awareness among the Malaysian population are being undertaken. Malaysia could view this as an opportunity to learn from Hong Kong in terms of effective advocacy activities in introducing ADs. Other potential advocacy options include communicating the availability of AD planning to patients during consultations or routine health checks. Clinical and public education in creating awareness among the population in partnership with patients is essential to achieving the intent of ADs. Both measures enable healthcare practitioners in anticipating patient needs and care management. A greater involvement of families and accounting for cultural sensitivity are necessary in improving knowledge of and perceptions to ADs (Ng et al. 2013). Equally important is the availability and provision of resources in equipping hospices and community care homes in implementing end-of-life care (Gendeh et al. 2016).

## Conclusion

This paper has highlighted the potential and challenges posed by AD legislation and examined their implications in multicultural Malaysia. The lack of clarity regarding the availability and legal status of ADs and the absence of consistent clinical guidelines has provided a timely opportunity to evaluate the feasibility of introducing a statutory framework to govern its use. In attempting to regulate the use of ADs through a statutory regime, we need to recognise that while statutes generally offer clarity in procedural matters, such outcomes are often achieved inadvertently at the price of invalidating an otherwise valid AD due to the failure to comply with formalities. The examples from Singapore, Hong Kong and England and Wales illustrate various similarities and differences in their approaches to ADs which are identifiable to the Malaysian context.

The ethical principle underpinning ADs presents a challenge distinct to the local framework; as such, to import such measures wholesale would be ill-conceived. Similarly, the influential roles of prevailing socio-cultural norms in medical decision-making affect the effective implementation of a statutory regime. Instituting a regulatory regime requiring formal written ADs underpinned by autonomy has unintentionally overlooked the special dynamics of patient-family-doctor relationships within these settings. A gradual introduction of the concept of ADs, fostered by creating awareness of its utility and providing relevant information to the public would encourage public awareness. The approach accommodates the needs of patients in using ADs in managing their healthcare within a multicultural society with strong family-oriented values and various religious beliefs, and circumvents the limitations of the statutory approach.

Additionally, it is equally important to consider the readiness of the healthcare services in offering and implementing ADs, taking into account the ways in which healthcare practitioners approach patients and their families in conversations surrounding ADs. A greater awareness and understanding of the issues pertaining to healthcare decision-making at the end of life, including planning for future care, ought to be widely recognised and disseminated. Public advocacy actions and education in terms of dispelling the myth regarding concepts of death, treatment withdrawal and advance decision-making are important and should precede any proposals for introducing AD laws.

## Footnotes

1 Cases that have come before the courts in England and Wales on ADs include *NHS Trust v T (adult patient: refusal of medical treatment)* [2004] EWHC 1279 (Fam); *HE v A Hospital NHS Trust & AE (by her litigation friend the Official Solicitor)* [2003] EWHC 1017 (Fam); *Re AK (Adult Patient) (Medical Treatment: Consent)* [2001] 1 FLR 129; *Re C (adult: refusal of treatment)*[1994] 1 All ER 819; *Re T (adult: refusal of medical treatment) *[1992] EWCA Civ 18; *NHS Trust v H W and others* [2005] 1 WLR 834 (CA); *A Local Authority v E (by her Litigation Friend the Official Solicitor), A Health Authority & E’s Parents* [2012] EWHC 1639 (COP). These examples are not limited to England and Wales; examples of AD disputes and treatment refusals in Australia and Canada include *Hunter and New England Area Health Service v A* (2009) 74 NSWLR 88; *Malette v Shulman* [1990] 67 D.L.R. (4th) 321.

2 For example, cases where Malaysian courts have followed the English courts in medical negligence disputes include *Hong Chuan Lay v Dr Eddie Soo FookMun* [2006] 2 MLJ 218;* Udaya Kumar A/L Karuppusamy v Penguasa Hospital Daerah Pontian* [2004] 2 MLJ 661; *AsiahBteKamsah v Dr Rajinder Singh* [2002] 1 MLJ 484; *Dr K S Sivananthan v Government of Malaysia* [2001] 1 MLJ 35.
